# The Association between the Concentration of Heavy Metals in the Indoor Atmosphere and Atopic Dermatitis Symptoms in Children Aged between 4 and 13 Years: A Pilot Study

**DOI:** 10.3390/children8111004

**Published:** 2021-11-03

**Authors:** Hyun Seung Choi, Michelle J. Suh, Sung Chul Hong, Ju Wan Kang

**Affiliations:** 1 Department of Otorhinolaryngology, National Health Insurance Corporation Ilsan Hospital, Goyang 10444, Korea; choihyunseung@gmail.com; 2 Department of Otorhinolaryngology, Jeju National University School of Medicine, Jeju 63243, Korea; myzetapotential@gmail.com; 3 The Environmental Health Center (Atopic Dermatitis & Allergic Rhinitis), Jeju National University, Jeju 63243, Korea; e_safejeju@jejunu.ac.kr; 4 Department of Otorhinolaryngology, Gangnam Severance Hospital, Yonsei University College of Medicine, Seoul 06237, Korea

**Keywords:** atopic dermatitis, heavy metal, lead, air pollutant

## Abstract

Background: A correlation between the harmful effects of air pollutants and atopic dermatitis has been reported. There are few studies on the correlation between the concentration of heavy metals in the indoor atmosphere and symptoms of atopic dermatitis. Methods: Twenty-two homes of children showing atopic dermatitis symptoms were enrolled, and eighteen homes with similarly aged children without symptoms or a history of atopic dermatitis participated as a control group. We measured the concentrations of various air pollutants (particulate matter 10, carbon dioxide, carbon monoxide, formaldehyde, nitrogen dioxide, volatile organic compounds (VOCs), ozone, radon, bacterial aerosols, and mold) as well as various heavy metals, such as lead, cadmium, and mercury, in the living room and children’s bedroom of each home. Results: Lead was more commonly detected in the indoor air in houses of children with atopic dermatitis (15/22) as compared to in the control group (3/18) (chi square test, *p* = 0.002). In adjusted logistic regression analysis, VOCs and lead were significantly associated with atopic dermatitis (*p* < 0.05). Conclusion: Our study shows that lead in indoor air might be associated with atopic dermatitis, even if the concentrations of airborne lead are below the safety levels suggested by health guidelines.

## 1. Introduction

Atopic dermatitis (AD) is a common inflammatory skin disorder in children, and its prevalence has been increasing worldwide [1]. Genetic and environmental factors have been suggested as the causes of AD. Both factors can influence the development of AD through two possible pathological mechanisms: an immunological imbalance between (T helper type 1 (Th1) and T helper type 2 (Th2) inducing a skin barrier defect, which could result in the development of AD, and the primary impairment of the skin barrier, which predisposes one to the penetration of allergens that cause the development of AD [2].

Among the various environmental factors, air pollutants have been examined in regard to their adverse effects on health. For instance, sulfur oxide compounds, nitrogen oxide compounds, carbon monoxide (CO), volatile organic compounds (VOCs), particulate matter (PM), toxic heavy metals, and radioactive pollutants such as radon are included in any discussion of dangerous air pollutants [2]. Exposure to these air pollutants can lead to respiratory diseases such as asthma, bronchitis, and allergic rhinitis in children [3,4]. In addition, air pollutants such as PM, CO, and VOCs can result in an increased risk of AD development and worsening of existing AD symptoms. Three-year-averaged concentrations of PM with diameters less than 10 μm (PM_10_), nitrogen dioxide (NO_2_), and CO have been significantly associated with the occurrence of lifetime eczema in children in France [5].

Morgenstern et al. showed a positive association between NO_2_ exposure and the risk of doctor-diagnosed eczema [6]. In addition, the concentrations of outdoor PM, toluene, and total VOCs were found to be higher on days when children reported severe AD symptoms than those on days when the children reported no symptoms [7].

Toxic heavy metals, such as cadmium, lead, and mercury, might be included in air pollutants related to painting, smoking, and automobile traffic. Kim et al. reported that prenatal cadmium exposure might be a risk factor in the development of AD in a six-month-old infant; however, the level of lead has not been associated with an increased risk of AD [8]. Although the harmful effects of toxic metals on the respiratory tract are well known, the effects of toxic heavy metals present in polluted air on the development of AD are not fully elucidated [9]. Therefore, this pilot study aim to investigate if the concentration of heavy metals in indoor air is higher in the homes of children diagnosed with AD compared to the homes of a control group.

## 2. Materials and Methods

### 2.1. Study Population

This study was conducted in Jeju and Seogwipo cities in Jeju island, South Korea. Applicants were recruited to measure indoor air quality at home. Twenty-two houses with children diagnosed with AD were recruited to measure indoor air quality at home. The presence of AD was confirmed using Hanifin and Rajka’s diagnostic criteria [10]. In addition, the indoor air quality of 18 children’s houses without history and symptoms of atopic dermatitis was also measured. All participants in the study have lived in their houses from birth. Air pollutant measurements were taken in the living rooms and in the subjects’ bedrooms during the daytime with the window remaining closed. Measurements were conducted from 11 June to 19 June 2010. Written informed consent was obtained from all participants, and the institutional review board of Jeju National University Hospital approved this study.

### 2.2. Measurement of Air Pollutant Concentration in Indoor Air

The measurement point was conducted at a height of 1.2 to 1.5 m from the floor at a location at least 1 m from the central wall (however, if there was a natural vent or mechanical ventilation system installed indoors, measurements were made at least 1 m from the supply port). Measurements were carried out under normal living conditions with the window closed so that outside air could not flow in.

We measured heavy metal concentrations (lead, cadmium, and mercury) in the indoor air. Heavy metals from room air were collected using cellulose filters (TE-230WH, Tisch Environmental, Inc., Cleveland, OH, USA) with a Zefon Escort ELF Personal Air Sampling Pump (Zefon International, Ocala, FL, USA). Lead, mercury, and cadmium levels were measured using graphite furnace atomic absorption spectrometry with Zeeman background correction (PerkinElmer AAnalyst 600, Turku, Finland).

In addition, we measured PM_10_, carbon dioxide (CO_2_), CO, formaldehyde, NO_2_, VOCs, ozone, radon, bacterial aerosols, and mold, as well as the temperature and humidity in each house. PM_10_ was measured using a mini-volume air sampler (Airmetrics Co, Eugene, OR, USA). CO and CO_2_ were sampled using an APEX personal air sampler (Casella, Bedford, UK), and their concentrations were analyzed using a nondispersive infrared spectrometer (GrayWolf™ IQ-610, Singapore; 300E CO analyzer, San Diego, CA, USA, respectively). Formaldehyde was sampled by 2,4-dinitrophenylhydrazine-coated sampling cartridges (SIBATA MP-100, Saitama, Japan) and was assessed using high-performance liquid chromatography (LC-10AD, Shimadzu, Tokyo, Japan). NO_2_ was analyzed with a chemiluminescent analyzer (Teledyne-API-200A, San Diego, CA, USA). VOCs were sampled by Tenax-TA (60/80 mesh; Supelco, Bellerfonte, PA, USA) and assessed using gas chromatography–mass spectrometry (GC–MS, Hewlett-Packard, San Jose, CA, USA). Ozone was measured using a Thornton M300 ultraviolet photometer (Mettler-Toledo, Columbus, OH, USA) with a wavelength of 254 nm. Radon was measured using Sun Nuclear 1027 (Sun Nuclear, Melbourne, FL, USA). Bacterial aerosols and airborne fungi were measured using KAS-110 (Kemik International, Seongnam, Korea) and a C-in incubator (Changshin, Seoul, Korea). Measurements of pollutants in the air were based on each manufacturer’s standard method.

### 2.3. Statistical Analyses

Each air pollutant concentration is presented as the mean value of concentrations measured in each living room and subject’s bedroom. Values of VOCs, mold, bacterial aerosol, ozone, formaldehyde, temperature, and humidity showed a normal distribution. PM_10_, CO, CO_2_, NO_2_, radon, and heavy metals (lead, mercury, and cadmium) did not show a normal distribution. The ^t^-test was used for variables with a normal distribution to compare the differences between the AD group and the control group. The Mann–Whitney test was used to analyze the difference in the median values between the AD group and the control group for variables that did not follow a normal distribution. An independent association between variables and AD occurrence was evaluated using logistic regression analysis. Adjusted logistic regression analysis was then performed, including a variable that showed *p* < 0.1 in unadjusted logistic regression analysis. Data (Supplementary File S1) were analyzed using SPSS 17.0 (SAS Institute, Cary, NC, USA). The *p* value was <0.05 and was considered statistically significant.

## 3. Results

Table 1 shows the demographic data of the study population.

The proportion of boys to girls was slightly higher in the AD group (16/22) compared to the control group (9/18); however, there was no statistical significance in the chi-square test. The average ages of the AD group and the control group were 7.7 years and 7.8 years, respectively, and hence, there was no significant difference. VOCs in houses with AD children were significantly higher than those in houses of the control group (*p* = 0.005). Mold levels in the air of houses with AD children were higher than those in houses without AD children, with a significance of *p* < 0.1. In addition, the humidity of indoor air was lower in houses with AD children than in houses without AD children, with modest significance (*p* < 0.1). On the other hand, PM_10_, CO, CO_2_, NO_2_, ozone, radon, bacterial aerosol, formaldehyde, and temperature showed no marked differences between the two groups.

**Figure 1:**
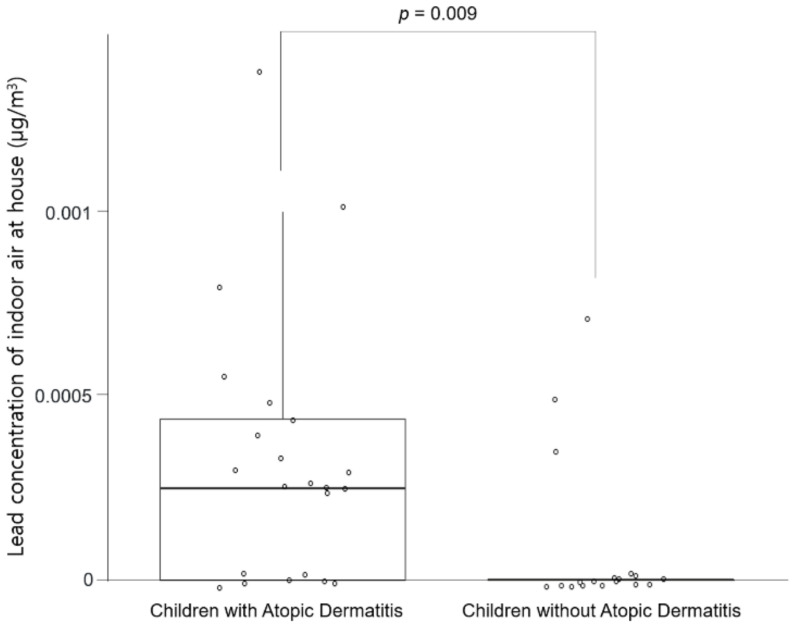
Differences in lead concentration in indoor air in the houses of children with and without atopic dermatitis (Mann–Whitney test).

Next, we analyzed the concentration of heavy metals in the indoor air. Lead in indoor air was detected in only three of the 18 houses in the control group. On the contrary, lead was detected in 15 of the 22 houses in the AD group. The odds ratio between the two groups was 10.7 in the chi-square test (*p* = 0.002). Cadmium in the air was detected in a total of 10 houses, four houses of children with AD, and six houses of children without AD. There was no statistically significant difference (*p* = 0.3). Mercury in the air was detected in a total of three houses (one house of children with AD and two houses of children without AD), and there was no statistically significant difference (*p* = 0.579). The Mann–Whitney test also showed that lead in indoor air in the AD group was significantly higher than that in the control group (Figure 1, *p* = 0.009). The Mann–Whitney test showed no statistical difference in the median value of cadmium and mercury between the AD group and the control group.

Finally, we tested the association between each variable and AD using unadjusted logistic regression analysis. As a result, we found that PM_10_, VOCs, mold, lead, and NO_2_ were correlated with AD with a significance of *p* < 0.1. Finally, independent association was analyzed using logistic regression analysis adjusted with variables, which showed *p* < 0.1 in unadjusted logistic regression analysis (Table 2). As a result, VOCs and lead were significantly associated with AD (*p* < 0.05).

## 4. Discussion

To the best of our knowledge, this is the first study whose results show that the lead levels in indoor air were higher in the houses of children with AD than in the houses of children without AD. There are findings based on the correlation of prenatal lead exposure with neurodevelopmental disorders [11,12]. In addition, there is a report on lead levels during pregnancy and the diagnosis of AD in infants [13], whose results are supported by the lead-induced altered immune responses and elevation of Immunoglobulin E (IgE) as determined in experimental studies. [14,15]. In relation to the results of this study, it can be considered that even a low level of exposure may affect AD susceptibility before and after birth and during childhood. Hon et al. established a connection between serum lead levels and the severity of eczema, quality of life, and atopy in children [16]. However, serum lead levels of most children were below the reference limit in their study. Moreover, the concentrations of airborne lead in all houses were lower than the suggested level in various guidelines [17]. Therefore, there remains uncertainty regarding the hazardous concentration of airborne lead that can affect AD.

The health effects of lead in air were shown to be less significant in children than in adults; airborne lead could be a major route of lead exposure for adults, while ingestion is more likely for children. The guidelines regarding airborne lead concentration levels remain inconsistent. Therefore, the guideline for lead concentration levels in the air was calculated using the level of lead concentration in the blood that can affect health. Lead at a level of 1 µg/m^3^ in air is approximately associated with 19 µg/L of lead in children’s blood. Blood lead levels should not exceed 100 µg/L in children, and average lead levels in indoor air should not exceed 0.5 µg/m^3^ on this basis [17]. In our study, the highest detected lead level in air was 0.0014 µg/m^3^. Although the standard for lead concentration in the atmosphere is not clear, our result shows the possibility that lead concentrations below the acceptable concentration can also affect atopic dermatitis.

Bohme et al. established that passive smoking is associated with an increased risk of eczema in four-year-old children [18]. Yi et al. also determined that childhood exposure to second-hand smoke is a major risk factor for the development of AD [19]. Previous studies have shown that serum lead levels increased in children aged 4–16 years who were exposed to second-hand smoke and that smoking could be an important source of airborne lead [20]. Our results postulate that the lead in cigarette smoke could lead to the development of AD. However, in this study, we did not take into consideration the smoking habits of the participating families. Further investigation into parental smoking habits may reveal more information about the relationship between lead levels in air and smoking.

In addition, the results of our study show that VOCs are also associated with AD. Previous studies have shown that various air pollutants are related to the development and severity of AD. Kim et al. reported that PM_10_, PM_2_._5_, toluene, and VOCs levels were higher on days when individuals had symptoms of AD than on days when they reported no AD symptoms [7]. Morgenstern et al. revealed a positive association between NO_2_ exposure and doctor-diagnosed eczema in German children [6]. In our study, PM_10_, VOCs, and NO_2_ were associated with AD with a significance of *p* < 0.1 in unadjusted logistic regression analysis, despite PM_10_ and NO_2_ not showing significance in adjusted analysis. Furthermore, mold was associated with AD in unadjusted analysis. Ukawa et al. found household mold to be a risk factor for the development of AD in boys; however, this association was not found in girls [21].

Our study has several limitations. First, we did not consider the allergic sensitization diagnosed by objective measurement. Allergic sensitization is one of the most critical features of AD, and, therefore, it should be taken into consideration. Second, as serum lead levels are the best indicator for lead exposure, we determined that an investigation considering serum lead levels would lead to accurate results. Third, the number of participants in the study population was too small, and a limited questionnaire was conducted. Detailed epidemiological and socioeconomic information of the subjects, including the total number of siblings with allergic symptoms and disease, family AD history, smoking, and parental education, was not available. Additionally, a one-time measurement of pollutant levels could lead to biased results. There was insufficient information on the degree of exposure to pollutants and the severity of symptoms at the time of measurement, and only one-time point measurement of the pollutant level was reviewed, so a study on the severity of symptoms according to the increase in the exposure period is needed. Moreover, it is necessary to consider the effect of ventilation as a confounding variable. When considering the indoor CO_2_ level as a reference value for ventilation [22], there was no difference between the two groups, so it could be estimated that they were in a similar ventilation environment. However, since each ventilation level was not completely identical, it is possible that this may have affected the results. Therefore, we believe that continual measurements of lead levels in a larger number of homes under controlled environments will lead to more accurate results. Finally, we did not investigate the severity of symptoms in this study. We believed that better conclusions might be drawn if the correlation between the severity of symptoms according to the concentration of each pollutant in the atmosphere was investigated.

## 5. Conclusions

Our study shows that lead in indoor air might be associated with atopic dermatitis symptoms, even if the recorded concentrations of airborne lead are below the reference level suggested by guidelines. However, our study provides only preliminary results, and further studies are needed to elucidate the risk of airborne lead on the development of atopic dermatitis in children.

## Figures and Tables

**Table 1:** Demographic data of study population.

Variables	Home with AD (*n* = 22)	Home without AD (*n* = 18)	*p*-Value
Sex (boy/girl) ^$^	16/6	9/9	0.194
Age (years) ^#^	7 (5–11)	7 (5–11)	0.946
Temperature (℃)	25.4 ± 1.0	24.9 ± 1.0	0.121
Humidity (%) *	74.8 ± 2.9	76.3 ± 2.4	0.099
PM_10_ (µg/m^3^) ^#^	25.7 (24.3–32.2)	25.2 (22.5–27.1)	0.155
CO_2_(ppm) ^#^	534.5 (455.4–637.6)	447.5 (386.5–563.5)	0.925
CO (ppm) ^#^	0.100 (0.100–0.100)	0.100 (0.100–0.113)	0.904
NO_2_ (ppm) ^#^	0.109 (0.083–0.145)	0.103 (0.082–0.163)	0.925
VOCs (µg/m^3^) *	227.3 ± 102.6	200.3 ± 55.8	0.005
Bacterial aerosol * (CFU/m^3^)	502.6 ± 240.3	437.4 ± 173.6	0.341
Mold (CFU/m^3^) *	59.4 ± 26.6	45.5 ± 20.1	0.078
Ozone (ppm) *	0.17 ± 0.01	0.17 ± 0.01	0.906
Radon (pCi/L) ^#^	0.200 (0.175–0.300)	0.200 (0.138–0.250)	0.527
Formaldehyde * (µg/m^3^) *	31.4 ± 17.2	27.9 ± 16.5	0.530

Abbreviations: AD, Atopic dermatitis; PM_10_, particulate matter with diameter less than 10 μm; CO_2_, carbon dioxide; CO, carbon monoxide; NO_2_, nitrogen dioxide; VOCs, volatile organic compounds. ^$^ Nominal variables: analyses using the chi-square test. * Variable with a normal distribution: presented as the mean with standard deviation and analyzed using a ^t^-test. ^#^ Variable did not show a normal distribution: presented as the median with interquartile range and analyzed using the Mann–Whitney test.

**Table 2:** Linear regression analysis considering the diagnosis of atopic dermatitis with dependent variables.

	*p*-Value	
Variables	Unadjusted	Adjusted ^#^
PM_10_ (µg/m^3^)	0.098 *	0.249
CO_2_ (ppm)	0.276	-
CO (ppm)	0.915	-
NO_2_ (ppm)	0.056 *	0.981
VOCs (µg/m^3^)	0.014 **	0.038 **
Bacterial aerosol (CFU/m^3^)	0.334	-
Mold (CFU/m^3^)	0.083 *	0.566
Ozone (ppm)	0.653	-
Radon (pCi/L)	0.274	-
Formaldehyde (µg/m^3^)	0.522	-
Lead (µg/m^3^)	0.028 **	0.035 **
Cadmium (µg/m^3^)	0.234	-
Mercury (µg/m^3^)	0.278	-
Temperature (℃)	0.125	
Humidity (%)	0.103	

* *p* < 0.1, ** *p* < 0.05. ^#^ PM_10_, NO_2_, VOCs, mold, and lead were included in analysis as confounding variables. Abbreviations: PM_10_, particulate matter with a diameter less than 10 μm; CO_2_, carbon dioxide; CO, carbon monoxide; NO_2,_ nitrogen dioxide; VOCs, volatile organic compounds.

## Data Availability

The raw data supporting reported results can be found in the Supplementary Materials Section.

## Supplementary Materials

The following are available online at https://www.mdpi.com/article/10.3390/children8111004/s1, File S1.
